# Systematic review and policy dialogue to determine challenges in evidence-informed health policy-making: findings of the SASHA study

**DOI:** 10.1186/s12961-021-00717-x

**Published:** 2021-05-04

**Authors:** Bahareh Yazdizadeh, Haniye Sadat Sajadi, Farideh Mohtasham, Mahsa Mohseni, Reza Majdzadeh

**Affiliations:** 1 Knowledge Utilization Research Centre, Tehran University of Medical Sciences, Tehran, Iran; 2 Knowledge Utilization Research Center, University Research and Development Center, Tehran University of Medical Sciences, Tehran, Iran; 3 Community Based Participatory Research Center, Knowledge Utilization Research Center, Tehran University of Medical Sciences, Tehran, Iran

**Keywords:** Evidence-informed policy-making, Evidence utilization, Health policy-making

## Abstract

**Background:**

Various interventions have been undertaken in Iran to promote evidence-informed health policy-making (EIHP). Identifying the challenges in EIHP is the first step toward strengthening EIHP in each country through the design of tailored interventions. Therefore, the current study was conducted to synthesize the results of earlier studies and to finalize the list of barriers to EIHP in Iran.

**Methods:**

To identify the barriers to EIHP in Iran, two steps were taken: a systematic review and policy dialogue. To conduct the systematic review, three Iranian databases and PubMed, Health Systems Evidence (HSE), Embase, and Scopus were searched. The reference lists of included papers and documentation from some local organizations were hand-searched. Upon conducting the systematic review, given the significance of stakeholders in clarifying the problem of EIHP, policy dialogue was used to complete the list previously extracted and to do advocacy. Selection criteria for the stakeholders included influential and informed individuals from knowledge-producing, knowledge-utilizing, and knowledge-brokering organizations. Semi-structured interviews were held with three important absent stakeholders.

**Results:**

Challenges specific to Iran that were identified included the lack of integration of the health ministry and the medical universities, lack of ties between health knowledge utilization organizations, failure to establish long-term research plans, neglect of national research needs at the time of recruiting human resources in knowledge-producing organizations, and duplication and lack of coordination in routine data obtained from surveillance systems, disease registration systems, and censuses. It seems that some challenges are common across countries, including neglecting the importance of inter- and intra-disciplinary studies, the capacity of policy-makers and managers to utilize evidence, the criteria for evaluating the performance of policy-makers, managers, and academic members, the absence of long-term programmes in knowledge-utilizing organizations, the rapid replacement of policy-makers and managers, and lack of use of evaluation studies.

**Conclusions:**

In this study, we tried to identify the challenges regarding EIHP in Iran using a systematic review and policy dialogue approach. This is the first step toward determining the best interventions to improve evidence-informed policy-making in each country, because these challenges are contextual and need to be investigated contextually.

**Supplementary Information:**

The online version contains supplementary material available at 10.1186/s12961-021-00717-x.

## Background

In spite of the global demand for the use of evidence in policy-making, the systematic use of research evidence is lacking [1]. It has been reported that although policy-makers utilize existing evidence in their decision-making, a gap still exists between access to scientific evidence and the systematic use of the evidence at different levels of the health system, including the policy-making process [2].

In Iran, various structural and procedural interventions have been undertaken to promote evidence-informed health policy-making (EIHP) [3]. The most important steps taken are the establishment of the Supreme Council of Health and Food Security (SCHFS) by the government as the main reference for health and food security policy-making and decision-making, in which evidence utilization has been defined throughout its policy-making cycle; establishment of the National Institute for Health Research (NIHR), aimed at producing evidence for the Ministry of Health and Medical Education (MOHME); creation of the Health Technology Assessment (HTA) programme [4]; and the development of standards and tariffs in the MOHME and the creation of the Health Policy-making Council within the MOHME. Nevertheless, the status of EIHP is weak.

A previous study examining research projects completed during 2007 and 2008 at six Iranian medical universities found that only 20% of them involved public health and health service research. Moreover, only 16% of relevant research had been utilized in developing documents related to policy-making [5]. Another study in Iran examined reports of HTAs conducted between 2008 and 2013 and indicated that all the reports revolved around medical equipment and drugs, and the remaining ministerial policies and plans had no HTA requests. Furthermore, this study showed that the greatest impact of HTA results involved decisions related to budget allocation for medical equipment [6].

It should be noted that although research evidence is quite convincing and plays a significant role in the policy-making process [7], in this manuscript we are not solely concerned with research evidence. Other types of evidence used in health policy-making include knowledge and information (acquired from consultations and polling from networks and groups, the internet, reports, and published documentation), interests and ideas (ideas and opinions of individuals, groups, and networks that are usually mixed with personal experiences, beliefs, values, and skills), political and economic circumstances (information related to state ordering, assessment of political risks, opportunities, crises, and available resources), routine health system data (such as surveillance system data, registration systems, and censuses), and survey data [8].

We decided to develop a road map of evidence-informed policy-making (EIPM) in Iran (the SASHA project), whose protocol has been published [9]. The first step of this project was to determine the challenges in EIPM in Iran. In this paper we will describe this part of the SASHA project.

Identifying the barriers and challenges in EIHP is the first step toward strengthening EIPM in the country, based on which targeted interventions can be designed. A number of general and common barriers in EIPM have been identified. For example, in 2016, Andermann et al. concluded that the most commonly discussed key themes regarding barriers included missing the window of opportunity, lack of contextualized evidence and uncertainty, controversial and conflicting evidence, and finally, conflict of interest, which are all associated with knowledge-producing organizations [10]. In 2013, Liverani et al. emphasized that the political system and institutional mechanism in knowledge-utilizing organizations and the political nature of health issues are the important factors influencing EIPM [11]. The characteristics of these barriers, which must be known in order to identify and tailor suitable solutions to address them, are context-specific and should be determined in each country.

Different studies have been conducted to identify the barriers to and facilitators of EIPM in Iran's health sector. Therefore, the current study was conducted to summarize the results of earlier studies and to finalize the list of barriers to EIPM in Iran's health sector. The latter was identified through a scoping review and policy dialogue.

## Methods

To identify the barriers to EIHP in Iran, two steps were taken: a systematic review and policy dialogue.

### Systematic review

The systematic review was conducted to identify the barriers to EIPM in Iran's health system. The inclusion criteria were primary and secondary studies conducted on EIPM in Iran with different research designs. The exclusion criteria included studies that examined the method for utilizing evidence and/or clinical decision-making or whose full texts were not accessible.

To conduct the systematic review, the following databases were searched for English-only literature, with no time limitation: international databases including PubMed, Health Systems Evidence (HSE), Embase, and Scopus, and Persian-language Iranian databases including Magiran, MEDLIB, Irandoc, and SID. To increase the sensitivity of the search, hand-searching was done in the reference lists of included papers and in the documentation from the related organizations in Iran, including the Parliament’s Research Centre, Social Security Organization Research Institute, the Deputy of Coordination's Policy-making Council, the Deputy of Planning, the Academy of Sciences, and the government legislatures. The search strategy is presented in Additional file 1: Appendix 1. We used keywords relevant to each of the concepts of evidence, policy-making, and evidence use, and specific interventions including HTA and guidance. All stages of the search and screening (primary and secondary) and data extraction were performed independently by two research team members. Data from the articles were extracted using a checklist containing the following items: article title, author's name, study objective and method, population and sample size, year of conduction, data collection tool, method of study analysis, and EIPM barriers. In the case of disagreement regarding the extracted data, a third team member examined the data. Next, data related to the extracted barriers underwent thematic analysis. Three main categories were deductively selected: knowledge-producing (PUSH), knowledge-utilizing (PULL), and knowledge-exchange organizations. The remaining categories and codes were inductively determined.

Because we wanted to identify the EIPM challenges in a single country, we decided to include all the identified relevant articles. Thus, the articles were not assessed for quality.

### Policy dialogue

Upon conducting the systematic review and extracting the barriers, given the significance of stakeholders in clarifying the problem [12], policy dialogue was used. The main goal of holding this meeting was to become familiar with the stakeholders' perceptions and opinions about barriers identified in the systematic review and to complete the list. Other objectives were to inform them of the status of evidence on the barriers to EIPM and to do advocacy. Selection criteria for the stakeholders included influential and informed individuals from knowledge-producing, knowledge-utilizing, and knowledge-exchange organizations. Forty-one participants were purposefully selected from knowledge-producing (14 persons), knowledge-utilizing (23 persons), and knowledge-exchange (four persons) organizations. They were informed of the goal of the meeting in advance by email or phone call. Once they agreed to participate, an invitation was sent along with a list of the barriers extracted in the systematic review. Three facilitators steered, led, and facilitated the policy dialogue. The meeting was 3 hours long. All the discussions were recorded upon obtaining consent. During this meeting, a list of the barriers extracted was given to the participants, and in addition to voicing their opinions, they were asked to complete the list. Afterwards, the meeting's discussions were transcribed. Those who were absent from the meeting were interviewed in person. The interviews were similarly recorded and later transcribed. The list of EIHP barriers extracted from the systematic review was finalized using the stakeholders' opinions.

The facilitators took notes during the meeting as well. To extract new barriers, the policy dialogue and interviews were analysed independently by two persons, and differences in opinion were examined during consensus meetings.

We used manifest content analysis [13] to extract new barriers (themes) from policy dialogue, by an inductive open coding process. We then compiled all barriers (from systematic review and policy dialogue) together in one coding list. In the categorization stage, we grouped related codes into subcategories, and we then grouped related subcategories into three main categories: evidence production organization, knowledge utilization organization, and interaction between knowledge producers and users.

## Results

**Fig. 1:**
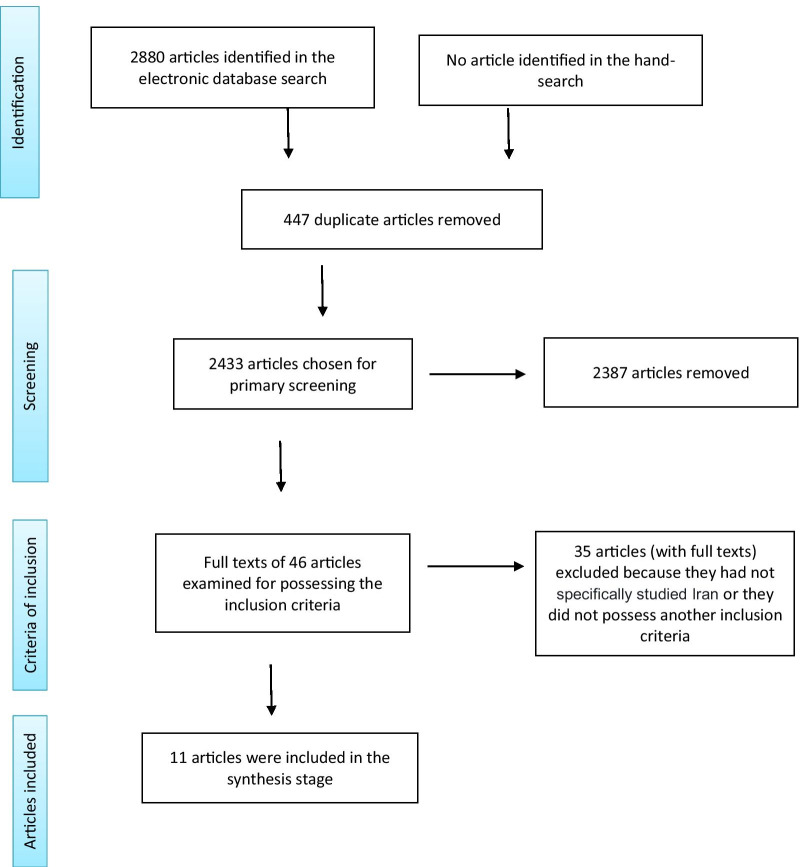
The screening algorithm based on PRISMA

Upon searching, we found 2880 articles, 2433 of which remained after deleting the duplicates. Following the secondary screening and examination of the articles' full texts, 11 articles relevant to the barriers and facilitators of EIPM in Iran's health system remained. A PRISMA [Preferred Reporting Items for Systematic Reviews and Meta-Analyses] flowchart [14] of the study's articles is illustrated in Fig. 1. Table 1 shows the list of articles included in the study and their characteristics.

**Table 1:** Characteristics of the included studies

Year published	Title	Objective	Study design	Participants
2014	Prioritizing Barriers to Successful Implementation of Hospital Information Systems [15]	To prioritize barriers to hospital information system implementation	Cross-sectional analytic-descriptive/questionnaire	24 participants; eight persons were clinical supervisors, seven were hospital managers, and nine were information technology department administrators
2013	Barriers of Clinical Practice Guidelines Development and Implementation in Developing Countries: A Case Study in Iran [16]	To identify the barriers to establishing production systems and applying the guidelines	Qualitative study with a thematic framework, in-depth interview, semi-structured, focus group discussion (FGD)	12 in-depth interviews with healthcare policy-makers and decision-makers, experts with previous experience in the production and adaptation of clinical practice guidelines, and evidence-based medicine education and development experts. FGD: 11 participants comprising healthcare policy-makers, managers, decision-makers
2014	Development of Evidence-Based Health Policy Documents in Developing Countries: A case of Iran [17]	To examine the barriers and facilitators of developing evidence-based health policy documents from the perspective of their producers in a developing country	Qualitative study with a framework analysis approach, semi-structured interviews, face-to-face interview, theory-based	23 producers of evidence-based policy documents in the MOHME
2014	Organizational Factors that Affect the Implementation of Information Technology: Perspectives of Middle Managers in Iran [18]	To find and review the organizational factors affecting information technology implementation in teaching hospitals of Tehran University of Medical Sciences	Cross-sectional descriptive, structured questionnaire	110 middle managers
2011	National policy-makers speak out: are researchers giving them what they need? [19]	The overall goal of this pilot study was to understand the perspectives and attitudes of policy-makers towards research, and towards using research to inform health policy across a spectrum of countries	Semi-structured, in-depth interviews, using a thematic-analysis approach	83 policy decision-makers in Argentina, Egypt, Iran, Malawi, Oman, and Singapore
2010	'Linking research to action' in Iran: Two decades after integration of the Health Ministry and the medical universities [20]	To examine the impact of integration of the health ministry and medical universities on “linking research to action” or “knowledge translation”	A qualitative study, a thematic framework, in-depth interview, FGDs	18 in-depth interviews and 10 FGDs with different stakeholders ranging from researchers, policy-makers, and service providers in medical and non-medical groups. In-depth interviews were held with policy-makers and decision-makers, and FGDs were held with researchers and practitioners
2008	Knowledge translation for research utilization: Design of a knowledge translation model at Tehran University of Medical Sciences [21]	To generate a model for knowledge translation in knowledge-producing units or organizations doing research	A narrative review (systematic reviews) and FGDs	650 articles and reports were reviewed. FGD: 23 researchers and decision-makers, ~10 researchers of medical universities, 5 managers of research institutes, 5 policy-makers from the MOHME, and 3 journal editors-in-chief, in three groups
2015	Implementation of Hepatitis Information Management System in Iran [22]	To design and implement an electronic hepatitis information management system to approach the mentioned limitations	Applied/developmental	Not reported
2014	How can we establish more successful knowledge networks in developing countries? Lessons learnt from knowledge networks in Iran [23]	To find the strengths and weaknesses of knowledge networks and to assess their effectiveness in Iran	Qualitative and quantitative, semi-structured, in-depth interviews, framework approach. The social network analysis approach was used to analyse effectiveness	In the qualitative section: 10 in-depth interviews were conducted with network directors and secretaries from 10 networks. In the quantitative section: research council members of six knowledge networks
2016	Stakeholder involvement in health technology assessment at national level: A study from Iran[24]	To evaluate the opinions of stakeholders on their roles in the HTA programme and to determine both the barriers and the facilitators in their organizations to help increase their collaboration in the HTA programme	Semi-structured interviews and policy dialogue A questionnaire The framework approach	10 representatives and 21 individuals participated in the policy dialogue
2016	Health information management system for elderly health sector: A qualitative study in Iran [25]	To investigate the current status of the health information management system in the field of geriatric health in Iran	Qualitative study A data collection form observed and reviewed by the researcher, interviewing experts, and faculty members, framework analysis	10 experts, managers, and faculty members

The final list of barriers is described separately for knowledge production, knowledge utilization, and knowledge exchange in Tables 2, 3, and 4, respectively. In each table, the barriers are ordered based on their identification sources: both systematic review and policy dialogue, only systematic review, and then only policy dialogue.

**Table 2:** The final list of EIPM barriers in knowledge production (PUSH), presented separately for the systematic review and policy dialogue

Barriers	Source	
A: Supportive processes	Review	Policy dialogue
Interactions between medical and non-medical universities have not been defined	*	
Organizational resources for information technology development are inadequate	*	
Academic members are selected regardless of the skills required	*	
Lack of mutual trust between researchers	*	
Researchers are dispersed, and there is no coordination among them	*	
There are no clearly defined task descriptions in knowledge translation (KT) units		*
The superficiality of policies and processes of teamwork thinking and interdisciplinary research		*
Researchers are not employed based on research needs		*
B: Incentive systems	Review	Policy dialogue
B1: Organizational values and goals		
Absence of a sustainable development approach in research		*
B2: Individual capacities and capabilities		
Researchers' lack of awareness on the necessity of KT	*	
Researchers' inadequate skills in research and KT methods	*	
Researchers' lack of familiarity with target audiences and the methodology of policy-making studies	*	
B3: Performance evaluation and reward programmes		
Considering quantitative criteria such as publication instead of giving importance to research quality and its applicability	*	
Neglecting KT activities in the performance evaluation	*	
Researchers' inadequate incentives to produce applied knowledge and the lack of the need to transfer their results	*	
Researchers' preference to choose easy instead of difficult research		*
Lack of incentives to interact with society		*
C: Characteristics of evidence	Source	
C1: Research evidence	Review	Policy dialogue
Weak strategic purchasing of research: research is not consistent with the users' needs and priorities	*	
Stakeholders do not participate in conducting the research	*	
Lack of trust of local evidence produced	*	
Absence of appropriate laws for protecting individuals' intellectual property rights	*	
The research results published are not up to date	*	
Local evidence is not used	*	
The persistence of journals' editor-in-chief councils on the publication of specific topics	*	
Poor quality of evidence		*
High volume of data or conflicting results, design, and differing values		*
Lengthy and conflicting review processes		*
C2: Routine health system data (registration, collection, analysis, dissemination)		
Those registering the routine data are unaware of the data's significance	*	
The lack of timely registration of patient data due to lack of coordination among different units and the unreliability of the data	*	
Incomplete implementation of health information systems	*	
Delay in or lack of decision-maker access to routine data, particularly data related to cost of services	*

**Table 3:** The final list of EIPM barriers in knowledge utilization (PULL), presented separately for the systematic review and policy dialogue

Barrier	Source	
A: The decision-making environment (macro-level and health sector)	Review	Policy dialogue
Absence of long-term plans and directors' lack of commitment to such plans	*	
Organizational, social, and political pressure in decision-making and the dominance of pressure groups over scientific evidence in policy-making	*	
Lack of communication between different sectors of the MOHME in the development and implementation of health policies	*	
Short tenure of policy-makers and their rapid replacement	*	
Directors are not chosen based on meritocracy	*	
Time limitations in organizational decision-making	*	
Personal interpretations of enforceable laws		*
Directors and policy-makers act based on their personal preferences		*
Evidence is exploited to approve a predetermined mental framework		*
Decision-makers' politicization		*
B: The health decision-making/policy-making process		
Lack of universality and institutionalization of the HTA process	*	
Absence of a specific criterion for prioritization and decision-making		*
Policies and programmes are not evaluated, and improvement is not made based on evaluation		*
No attention is paid to the contextualization of interventions		*
Panels of experts are used instead of research, and the panels are not held properly		*
Solutions are presented without complete and comprehensive data backup		*
C: Supportive processes and structures		
Lack of supervision, rules, and regulations regarding the development and implementation of guidelines	*	
Structural, financial, and legislative limitations in ordering the research needed	*	
Lack of processes that enforce the use of evidence in decision-making		*
Lack of support of senior policy-makers (e.g. Parliament representatives) by scientific groups		*
Shortage of skilled human resources for evidence utilization		*
D: Incentive system		
D1: Organizational and individual goals and values		
Absence of political support for evidence utilization in decision-making	*	
Policy-makers' inappropriate perceptions of the need for evidence utilization/ Decision-makers do not feel the need to utilize scientific evidence	*	
The health ministry's health decision-makers' preference to produce evidence themselves	*	
Giving priority to personal or organizational preferences over evidence	*	
Lack of health decision-makers' trust in the local research evidence	*	
Lack of commitment to evidence utilization in decision-making		*
Policy-makers' inappropriate perceptions of the real outcomes of policy execution		*
The perception of evidence utilization as a luxurious tool rather than strengthening and improving the health system		*
Lack of decision-maker transparency and accountability		*
D2: Individual capacities and capabilities		
Policy-makers' lack of awareness and skills in the analysis and rapid utilization of evidence	*	
Inappropriateness of individuals' skill and knowledge for policy-making and management; absence of strategic thinking among decision-makers		*
Superficial and simplistic knowledge regarding issues, problems, and solutions		*
D3: Performance evaluation and reward programmes		
Inappropriateness of indices for managers' performance evaluations (there's a quantitative approach, and the number of decisions is important); There is no criterion for evidence utilization in the managers' evaluation	*	*
The supervision and evaluation system of decision-makers is not evidence-based		*
The noncompetitive advantage of evidence utilization among policy-makers and managers and negative attitude towards policy-makers and managers who utilize evidence		*

**Table 4:** The final list of EIPM barriers in the interaction between knowledge producers and users, presented separately for the systematic review and policy dialogue

Barrier	Review	Policy dialogue
A: Supportive process		
Research priorities are not identified based on evidence of users' needs or by the administrative field	*	*
Lack of sufficient tools for identifying problems	*	
Research results are not actively disseminated and/or they are disseminated inappropriately	*	
Research results are not published in the users' appropriate language	*	
Political barriers in publishing special research results	*	
B: The communication system		
Lack of interaction between knowledge producers and users/administrative fields	*	
As a bridge between knowledge producers and users, the research network plan has not been completely executed	*	
Conflicts of interest between knowledge producers and decision-makers	*	
Lack of trust between knowledge producers and decision-makers	*	
The context of medical education integration has not been used to create a link between the university and the community		*
C: The information system of the knowledge translation process		
Absence of an appropriate database for identification of researchers	*	
Absence of an appropriate databank for accessing research results	*	
Inappropriate situation in data and information sharing in the system		*
D: The supportive procedures of the knowledge translation process		
Absence of an appropriate financial arrangement to produce the scientific evidence required	*	
Inadequate financing of knowledge-dissemination activities	*	
Absence of a clear-cut framework for applying a research result as evidence	*	
Inefficiency of the health education system to help produce and uptake evidence		*

## Discussion

The aim of this study was to identify EIPM challenges in Iran to determine effective measures toward promoting EIHP by undertaking appropriate interventions aimed at mitigating these challenges.

The differences found by comparing the barriers we identified with those of international studies underscores the fact that this identification process should be conducted individually for every country; although some of these barriers are common to all countries, others are specific to each.

We found three systematic reviews conducted across the globe in 2002, 2011, and 2014 [2, 26, 27] that examined EIPM barriers. The most commonly identified barriers in these studies were a lack of timely access to relevant evidence; lack of transparent, high-quality, and interpretable evidence; lack of communication and mutual trust between researchers and policy-makers; and policy-makers' lack of research skills, power struggle, and financial issues. Although we identified the barriers mentioned in these three systematic reviews, we identified further barriers that are specific to Iran's context and others that can be applied to various contexts as well, but which may have been addressed less in other studies.

The specificity of some barriers to certain contexts is important because it emphasizes the significance of choosing interventions appropriate to the EIPM status. A study published in 2017 specifically noted that important EIPM barriers differed in six European countries [28].

One such barrier specific to Iran is the failure to exploit the capacity created by the medical education integration in 1985, which was followed by the exclusion of medical universities and faculties from the Ministry of Science and their inclusion in the MOHME. By bringing these aforementioned systems (health care, medical education, and health research) closer together, integration could have potentially led to an intertwining of education, research, service delivery, and relevant policy-making. Thus, the needs of the service delivery system could have given rise to research questions and consequently to learning. However, this potential has yet to be converted into a sustainable and universal act.

Another important identified challenge is the lack of ties between the various sectors of the MOHME; different sectors collect different health-related data that may be appropriate for evaluating the policies of other sectors. However, due to the lack of communication between these sectors and lack of transparency regarding intellectual rights, multiple parallel measures are taken, which affects the efficiency of the health system as a whole and specifically with respect to EIPM.

One other profound challenge in Iran is the failure to address long-term research plans. As a result, the policy-maker is constantly faced with questions which lack evidence-based responses at the time of need. Clearly, to obtain evidence-based answers and resolve future issues, we must identify research needs now and begin to produce the required evidence. This is the only way that the gap between the policy-maker's question and access to evidence can be mitigated. To this end, long-term planning is needed for conducting research and producing evidence.

One more important challenge is the inappropriate criteria for evaluating the performance of policy-makers and managers, as well as academic members, which dissuades them from supporting EIPM. Policy-makers and managers are evaluated by the quantity of programmes and not the extent of evidence use in developing those programmes. Similarly, academic members and organizations are evaluated by their numbers of published articles and are promoted accordingly. Although these systems also assign scores to the use of research results in policy-making, they ignore the fact that multiple factors are involved in policy-making, and evidence and research results are only one part [29]. Moreover, the researcher does not play a major role in the utilization of evidence in policy-making, so it should not be considered as a criterion in evaluating their performance. Here, if a researcher chooses their research based on need and conducts high-quality research and produces a clear message, they must receive a score equal to that of a published article. However, such an approach does not yet exist yet in the evaluation of knowledge-producing organizations and individuals. All these issues push researchers toward choosing research topics that have a greater likelihood of being published in international journals over a shorter period of time. Given the lower likelihood of publication of articles derived from health policy and systems research that actually reflects the current research needs of the country, researchers are less inclined to conduct such research studies. Moreover, the policy-makers and managers in the MOHME are also academic members, who must go through the steps of promotion—which is very dependent on article publication—alongside their health policy-making and management. This will specifically encourage these individuals to conduct research themselves while in their managerial positions, and in turn prevent them from interacting with other researchers and thereby prolonging the duration of evidence production, all of which have negative effects on EIPM.

A specific challenge in Iran is the neglect of national research needs at the time of recruiting human resources in knowledge-producing organizations. In Iran, knowledge production takes place in universities and their affiliated research centres, which have two major traits: their research resources are governmental (which are usually allocated annually and are constantly susceptible to cuts and unsustainability), and the recruitment of educational and research staff is such that when a person enters the system, they remain in that educational or research position as an academic member until they retire. Therefore, in this system, human resources should be recruited based on the country's needs, given that health and health policy and systems research have been neglected, and service delivery and educational needs are always given priority over research needs. Furthermore, the number of research human resources in the necessary fields needs special attention.

Further barriers were identified in our study that have been addressed to a lesser degree in studies in other countries, such as the importance of inter- and intra-disciplinary studies in strengthening EIHP. Health-related difficulties and outcomes are complex issues that need to be resolved by answering research questions through various disciplines and fields, including health economy, behavioural change studies, and health promotion. In fact, an actionable message (which must clearly specify who should do what, when, and how to resolve an issue) cannot be produced by a single research study. Moreover, research on EIPM covers a range of disciplines such as public health, political philosophy, behavioural science, public policy, and administrative science [10]. These two subjects underscore the significance of focusing on the promotion of inter- and intra-disciplinary studies. It must be noted that in Iran, medical and non-medical universities are separate entities (the first is under the supervision of the MOHME, and the second under the supervision of the Ministry of Science). Therefore, their policy-making is done in two different ministries, and even their physical environments are separate. Thus, the promotion of inter- and intra-disciplinary studies must be associated with the undertaking of certain interventions to proximate education and research in medical and non-medical universities.

Routine data obtained from surveillance systems, disease registration systems, and censuses are very good sources for evidence production in health policy-making. And since they are routinely produced, the time gap between the need for evidence and the availability of potential evidence can be very short. Nevertheless, parallelism and poor coordination still exist in this field. Hence, data may be collected by different sources that have different results. Some data are still not collected in the country, their accuracy and quality are not appropriately assessed, and finally, they are not available at the right time. Therefore, any intervention and investment aimed at improving data collection will have a profound effect on strengthening EIPM.

The researchers' individual capacity to conduct quality research and to produce a clear message appropriate to the target audience and the policy-makers' and directors' individual capacity to utilize evidence are important barriers that need interventions. However, interventions cannot be designed without taking into account the context. To eliminate this barrier, we must know the individual capacity of technical experts to determine to what extent the evidence development should be carried out by user organizations (technical experts). This limitation must be kept in mind when designing an intervention in a country where—for any reason—experts from its evidence-utilizing organizations cannot produce quality evidence.

The absence of long-term programmes in knowledge-utilizing organizations and the rapid replacement of managers have negative impacts on EIPM, as evidence production cannot keep up with the pace of programme and manager changes. Thus, appropriate interventions must be designed to institutionalize the existing processes of decision-making organizations in a manner that reduces the impact of personal preferences on policies (their design and implementation).

One barrier that has been a focus of attention in other countries as well is the lack of policy evaluation and/or lack of use of evaluation studies’ result for policy improvements [30]. Interventions with the aim of increasing implementation of evaluation studies and policy analysis results have an important role in strengthening EIPM in countries where policy-making is mostly top-down.

Mistrust in the quality of local evidence—be it right or wrong—is a major factor impeding the localization of health policies and programmes. Lack of stakeholder participation in conducting the research aggravates the situation and also leads to research results that are irrelevant to the actual policy-making circumstances.

Here, the barriers and challenges of EIPM were classified into three components: push, pull, and exchange. Nevertheless, given that health system structures differ in different countries, the solutions for eliminating these barriers are different. In Iran, where health service delivery is integrated with education and research, the governance of undertaking relevant interventions occurs in a single ministry. However, it is a different story in countries where these components are separate. What matters is that the health system constitutes a complex system; to remove these barriers, we must consider a systemic approach, which means that multiple interventions at various individual and organizational levels—push, pull, and exchange—must be conducted simultaneously and over time [11]. This issue was discussed as an emerging theme, “developing comprehensive and coherent systems promoting implementation to impact”, in the 2019 *In the Trenches: Implementation to Impact International Summit* [31].

To choose interventions appropriate to the barriers identified, the best approach would be to identify the barriers which have the greatest impact and/or the greatest affect on other barriers, which is not easily determined. We are faced with a complex system in which the causes and effects are intertwined, and constructing a causal network for these barriers is not possible due to the paucity of experimental studies conducted in this domain.

We must remember, though, that the removal of some of these barriers is an undeniable *reality*, such as increasing the quality and relevance of evidence and its timely availability. There is no need to determine the cause and significance of these barriers; their removal is *requisite*. In 2017, Hanney et al. reviewed 36 multi-project research programmes and recommended fundamental solutions including discussions of research topics with users, the best way to conduct the research, and the necessary mechanisms in place for receipt and use of research [32]. Countries should try to find the best way to implement these solutions in this context: the SASHA project is one example.

The selection of interventions aimed at removing the barriers identified in each country must take into account the existing facilitators. There are appropriate structural facilities aimed at increasing interaction between knowledge producers and users in Iran. Some of these facilitate exchange between the MOHME and the universities, such as the NIHR, the HTA office, and knowledge management units in medical universities. Others are present within policy-making organizations, like the Higher Insurance Institute, and yet others have been established in medical universities, such as the Centre for Academic Policy Research and Development of Tehran University of Medical Sciences. From the EIPM perspective, the assessment of each of these structures and elimination of their barriers are important steps forward.

Finally, one of the important actions which will ensure the sustainable improvement of EIPM in each country is that of a systemic approach. This means that it should be done in two directions, impact assessment of health research according to health and socioeconomic impact, and utilization of evidence in policy-making [33].

### Strengths and limitations of the study

The strength of the current study is its methodology of barrier identification. Barriers were identified through systematic review and policy dialogue, the results of which indicated that the latter was very important in completing the list of barriers.

In 2017, Cairney et al. noted important limitations in studies on EIPM barriers, including a lack of policy theory, restricted data collection methods (e.g. interviews with minimal knowledge of policy process), and ignoring the role of values in politics [34]. Our study suffers from these limitations because of the presence of these limitations in the primary studies included, although in three of the primary studies, researchers used some theories or presumptions for identifying the barriers, although they do not exactly comport with policy theory.

Another limitation of the primary studies is that the influence of the specific type of policy was not investigated, and so we were not able to discuss it. As described by Mulgan in 2003, the policy field is an influential factor in the use of knowledge: in “stable policy fields”, where knowledge is settled, there is effective use of knowledge in policy, whereas in “policy fields in flux” (where the knowledge is contested and disagreement exists) and “inherently novel policy fields” (where there is no existing base of knowledge), the use of evidence in policy-making is weak [35]. The studies included in this scoping review investigated barriers to EIPM in hospital information systems, information technology, a hepatitis and elderly information management system, clinical practice guidelines, HTAs, evidence-based health policy documents, and knowledge networks, and three papers investigated general fields. In these primary studies, the policy fields were not specified; thus we were not able to discuss the influence of the policy field and type of evidence in EIPM.

Another limitation which is inherent in EIPM and emerges from the complexity of health systems and health research systems is the difficulty in prioritizing barriers in each part of push, pull, and exchange. It is not easy to map the causality network of barriers, and there is no means of estimating the effect of each barrier.

Finally, we did not include a critical appraisal of the primary studies. The objective of critical appraisal is to identify selection and information bias and its impact on the results and interpretation of the results. In the present review, if we had excluded studies because of selection bias, it would have reduced the comprehensiveness of the list of challenges. Therefore, even though the barriers identified from stakeholders in the studies may not have been comprehensive, it was not rational to exclude them. The impact of information bias would include challenges incorrectly identified or incomplete lists of challenges, but even if we knew the type and the source of information bias in the primary studies, there was still no basis for including some challenges and excluding others. For this reason, we did not critically appraise the primary studies, although we should note that there may have been some challenges that were not unidentified.

## Conclusion

In this study, we tried to identify the challenges in EIPM in Iran by using a systematic review and policy dialogue. This is the first step in choosing the best interventions to improve EIPM in each country, because these challenges are contextual and need to be investigated contextually. The next steps will be to find the best solutions to each of these challenges by considering international experience and context.

## Supplementary Information

Additional file1 (DOCX 14 KB)

## Data Availability

All data generated or analysed during this study are included in this published article and its supplementary information file.

## Abbreviations

EIHP: Evidence-informed health policy-making
NIHR: National Institute for Health Research
MOHME: Ministry of Health and Medical Education
HTA: Health technology assessment
PRISMA: Preferred Reporting Items for Systematic Reviews and Meta-Analyses
FGD: Focus group discussion
