# Supplementary material for: Systematic review and policy dialogue to determine challenges in evidence-informed health policy-making: findings of the SASHA study
Source: Health Res Policy Syst. 2021 May 4;19:73. doi: 10.1186/s12961-021-00717-x (PMC8097912; doi:10.1186/s12961-021-00717-x)
Supplement: Supplementary file 1 — Additional file1 (DOCX 14 KB) [file 12961_2021_717_MOESM1_ESM.docx]

**Supplementary file 1: Search strategy in PubMed**

1. health care policy[Title/Abstract]
2. decision making[Title/Abstract]
3. health polic*[Title/Abstract]
4. policy decision*[Title/Abstract]
5. policy mak*[Title/Abstract]
6. policymak*[Title/Abstract]
7. public polic*[Title/Abstract]
8. health plan*[Title/Abstract]
9. Policy[MeSH Terms]
10. Health Policy[MeSH Terms]
11. Decision Making, ganizational[MeSH Terms]
12. organization and Administration[MeSH Terms]
13. Administrative Personnel[MeSH Terms]
14. Health Planning[MeSH Terms]
15. policy-mak*[Title/Abstract]
16. policy[Title/Abstract]
17. policies[Title/Abstract]
18. policy analys*[Title/Abstract]
19. policy analyz*[Title/Abstract]
20. Implementation Plan[Title/Abstract]
21. strategy[Title/Abstract]
22. regulation[Title/Abstract]
23. Health program[Title/Abstract]
24. Road map[Title/Abstract]
25. master plan[Title/Abstract]
26. practice guideline [Title/Abstract]
27. adaptation[Title/Abstract]
28. execution[Title/Abstract]
29. quality improvement[Title/Abstract]
30. Enact[Title/Abstract]
31. agreement[Title/Abstract]
32. health technology assessment[Title/Abstract]
33. policy brief[Title/Abstract]
34. guidance[Title/Abstract]
35. policy dialogue[Title/Abstract]
36. policy document[Title/Abstract]
37. law[Title/Abstract]
38. protocol compliance[Title/Abstract]
39. development[Title/Abstract]
40. developer*[Title/Abstract]
41. provision[Title/Abstract]
42. provider*[Title/Abstract]
43. health care delivery[Title/Abstract]
44. health* manag*[Title/Abstract]
45. health care management[Title/Abstract]
46. service provi*[Title/Abstract]
47. evidence*[Title/Abstract]
48. research*[Title/Abstract]
49. science*[Title/Abstract]
50. scient* [Title/Abstract]))
51. infmation*[Title/Abstract]
52. data*[Title/Abstract]
53. knowledge*[Title/Abstract])
54. innovation*[Title/Abstract]
55. barrier*[Title/Abstract]
56. facilitat*[Title/Abstract]
57. uptake*[Title/Abstract]
58. utilis*[Title/Abstract]
59. utiliz*[Title/Abstract]
60. health care utilization[Title/Abstract]
61. diffus*[Title/Abstract]
62. disseminat*[Title/Abstract]
63. adapt*[Title/Abstract]
64. adopt*[Title/Abstract]
65. implement*[Title/Abstract]
66. aid[Title/Abstract]
67. aids[Title/Abstract]
68. aiding[Title/Abstract]
69. aided[Title/Abstract]
70. assist*[Title/Abstract]
71. bar[Title/Abstract]
72. barred[Title/Abstract]
73. barring[Title/Abstract]
74. block*[Title/Abstract]
75. hinder*[Title/Abstract]
76. hindrance*[Title/Abstract]
77. impede*[Title/Abstract]
78. impeding[Title/Abstract]
79. impediment*[Title/Abstract]
80. obstruct*[Title/Abstract]
81. promot*[Title/Abstract]
82. restrain*[Title/Abstract]
83. restrict*[Title/Abstract]
84. strengths[Title/Abstract]
85. weaknesses[Title/Abstract]
86. opptunities[Title/Abstract]
87. threats[Title/Abstract]
88. determinant[Title/Abstract]
89. pitfall[Title/Abstract]
90. process[Title/Abstract]
91. shortcoming[Title/Abstract]
92. limitation[Title/Abstract]
93. infrastructure[Title/Abstract]
94. OR/ 1 to 46
95. OR/ 47 to 54
96. OR/ 55 to 93
97. 94 AND 95 AND 96
